# An Evolutionary Conserved Role for Anaplastic Lymphoma Kinase in Behavioral Responses to Ethanol

**DOI:** 10.1371/journal.pone.0022636

**Published:** 2011-07-22

**Authors:** Amy W. Lasek, Jana Lim, Christopher L. Kliethermes, Karen H. Berger, Geoff Joslyn, Gerry Brush, Liquan Xue, Margaret Robertson, Monica S. Moore, Karen Vranizan, Stephan W. Morris, Marc A. Schuckit, Raymond L. White, Ulrike Heberlein

**Affiliations:** 1 Department of Neurology, Ernest Gallo Clinic and Research Center, University of California San Francisco, Emeryville, California, United States of America; 2 Departments of Pathology and Oncology, St. Jude Children's Research Hospital, Memphis, Tennessee, United States of America; 3 Functional Genomics Laboratory, University of California, Berkeley, California, United States of America; 4 Department of Psychiatry, University of California San Diego, La Jolla, California, United States of America; 5 Department of Anatomy and Program in Neuroscience, University of California San Francisco, San Francisco, California, United States of America; Alexander Flemming Biomedical Sciences Research Center, Greece

## Abstract

Anaplastic lymphoma kinase (*Alk*) is a gene expressed in the nervous system that encodes a receptor tyrosine kinase commonly known for its oncogenic function in various human cancers. We have determined that *Alk* is associated with altered behavioral responses to ethanol in the fruit fly *Drosophila melanogaster*, in mice, and in humans. Mutant flies containing transposon insertions in *dAlk* demonstrate increased resistance to the sedating effect of ethanol. Database analyses revealed that *Alk* expression levels in the brains of recombinant inbred mice are negatively correlated with ethanol-induced ataxia and ethanol consumption. We therefore tested *Alk* gene knockout mice and found that they sedate longer in response to high doses of ethanol and consume more ethanol than wild-type mice. Finally, sequencing of human *ALK* led to the discovery of four polymorphisms associated with a low level of response to ethanol, an intermediate phenotype that is predictive of future alcohol use disorders (AUDs). These results suggest that *Alk* plays an evolutionary conserved role in ethanol-related behaviors. Moreover, *ALK* may be a novel candidate gene conferring risk for AUDs as well as a potential target for pharmacological intervention.

## Introduction

Alcohol use disorders (AUDs) are a group of devastating conditions with serious health and social consequences. The World Health Organization estimates that 76.3 million people have an AUD and that each year the harmful use of alcohol kills 1.8 million people (WHO Global Status Report on Alcohol 2004). Genetic and environmental factors contribute to the development of AUDs. The heritability for alcohol dependence is approximately 0.5, indicating a strong genetic component for predisposition to this disease [1], [2]. Several genes have been linked to alcohol dependence and behavioral responses to alcohol, including *COMT*, which is involved in catecholamine metabolism, *ALDH2* and *ADH1B*, enzymes involved in alcohol metabolism, and several GABA_A_ receptor subunits [3]. However, the genetics of AUDs remain poorly understood and the predisposition to AUDs likely involves many genes. We have employed an invertebrate model organism, the fruit fly *Drosophila melanogaster*, to identify novel genes that modify the behavioral response to ethanol [4]. This approach has been successfully used to identify candidate genes that regulate not only acute behavioral responses to ethanol but also alcohol consumption in mammals [5].

Here, we describe the identification of anaplastic lymphoma kinase (*ALK*) as a candidate gene for AUDs based on evidence from flies, mice, and humans. ALK is a receptor tyrosine kinase in the insulin receptor family that was first identified as an oncogenic chromosomal translocation in anaplastic large cell lymphoma [6]. More recently, translocations and mutations in *ALK* have been associated with lung cancer [7] and neuroblastoma [8], [9], [10], [11], suggesting a key function for *ALK* in the development of several cancers. With regard to the nervous system, *Drosophila Alk (dAlk)* is important for axon targeting in the retina and synapse development at the neuromuscular junction [12], [13]. In mice, *Alk* is expressed in the developing and adult nervous system [14], [15] and inhibits hippocampal progenitor cell proliferation as well as depression-associated behaviors [16]. One human study has shown an association of polymorphisms in *ALK* with schizophrenia in a Japanese population [17], suggesting that *ALK* potentially affects the development of psychiatric disorders.

We have identified *dAlk* as a transcript regulated by *Drosophila* LIM-domain only (dLMO) using gene expression microarrays. Previous studies implicated *dLmo* and the mammalian homologs *Lmo3* and *Lmo4* in behavioral responses to alcohol and cocaine [18], [19], [20]. We therefore hypothesized that *Alk* function might also regulate behavioral responses to these drugs of abuse. Here, we provide data supporting this hypothesis using experimental approaches that test ethanol-related behaviors in flies, mice, and humans. We found that *Alk* regulates the acute sedating effect of ethanol in flies and mice as well as ethanol consumption in a binge-drinking model in mice. In humans, polymorphisms in *ALK* were found to be associated with a low level of response to alcohol in two measures, body sway and subjective high, two phenotypes that predict future AUDs [21], [22]. Together, these data suggest that *ALK* is a candidate gene predisposing individuals to a higher risk for developing AUDs.

## Results

### *dAlk* expression is regulated by *dLmo* and modulates ethanol-induced sedation in *Drosophila*

**Figure 1:**
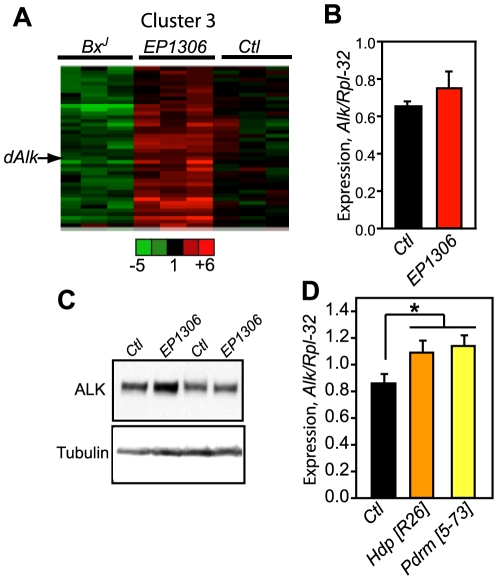
*dLmo* regulates expression of *dAlk*. (A) Microarray analysis of *dLmo* gain-of-function (*Bx^J^*), loss-of-function (*EP1306*), and control flies (Ctl, *w;iso*). Three samples of RNA from fly heads of each genotype were hybridized to Affymetrix Drosophila 2.0 oligonucleotide microarray chips and subjected to HOPACH clustering after data processing and normalization. Shown is cluster 3, containing 43 genes showing an increase in *dLmo* gain-of-function and decrease in loss-of-function mutants. The position of *dAlk* in the cluster is indicated by an arrow. Green color indicates decreased expression and red increased expression (fold change) compared to the control. *dAlk* expression changes were significant by ANOVA (*P* = 0.015) (B) qPCR showing an 18% increase in *dAlk* expression in flies carrying the *EP1306* allele. Total RNA was isolated from whole flies and cDNA synthesized for analysis by PCR. Expression of the *dAlk* transcript was normalized to the control transcript *Rpl-32*. (C) Increased ALK protein expression in *EP1306* fly heads compared to control fly heads (*Ctl*) by western blotting. ImageJ quantification of the blots and normalization to α-tubulin protein levels indicated an overall 20% increase in ALK in *EP1306* fly heads. (D) qPCR indicating increased *dAlk* expression in *dLmo* loss-of-function mutant flies *Hdp* and *Pdrm*. *P = 0.05 by ANOVA, n = 6–7 independent biological replicates.

Recently we found that the *Drosophila* LIM-domain only (dLMO) transcriptional regulator controls sensitivity to ethanol-induced sedation [20]. To identify novel transcriptional targets of dLMO that might mediate its effects on behavioral responses to ethanol, we performed gene expression microarray analysis of flies carrying either a *dLmo* loss-of-function mutant, *EP1306* [18], or a gain-of-function mutant, *Bx^J^* [23], [24]. We discovered 555 genes, out of 18,952 surveyed, whose expression was significantly altered in one or both of the *dLmo* mutants compared to control flies (Table S1). The 555 genes were clustered using the HOPACH algorithm [25], resulting in 7 clusters. Since the classically described function of *dLmo* is negative transcriptional regulation of genes [26], we focused on cluster 3, containing 43 transcripts exhibiting increased expression in the *dLmo* loss-of-function and decreased expression in the *dLmo* gain-of-function mutant. The gene encoding the fly homolog of anaplastic lymphoma kinase (*dAlk*), encoding a receptor tyrosine kinase in the insulin receptor superfamily, was a member of this cluster (Figure 1A). *dAlk* expression was decreased by 14% in *Bx^J^* flies and increased by 26% in *EP1306* flies. We did not observe any changes in the expression of the *dAlk* ligand *Jeb* in the *dLmo* mutant flies, suggesting a specific effect on *dAlk* expression and not other members of this pathway (data not shown). To confirm the microarray results, we examined *dAlk* expression using quantitative real-time PCR (qPCR) in *EP1306* and *Bx^J^* flies. *dAlk* expression was increased by 18% in the *EP1306* flies, consistent with the microarray results (Figure 1B). However, we were unable to recapitulate the small decrease in *dAlk* expression in *Bx^J^* flies observed in the microarray study (data not shown). It is possible that levels of dLMO are sufficiently high such that an additional increase in the *Bx^J^* mutants would not significantly affect *dAlk* expression. ALK protein levels were also increased by 20% in the heads of *EP1306* flies when examined by western blotting (Figure 1C), indicating that ALK protein levels changed with a similar magnitude as the transcript in the *EP1306* mutant. We next examined *dAlk* RNA expression in flies carrying additional *dLmo* loss-of-function alleles, *Hdp* and *Pdrm* [18] and found that *dAlk* expression was indeed increased by ∼30% in the *dLmo* mutants compared to control flies (Figure 1D), also consistent with the expression data in the *EP1306* mutant.

**Figure 2:**
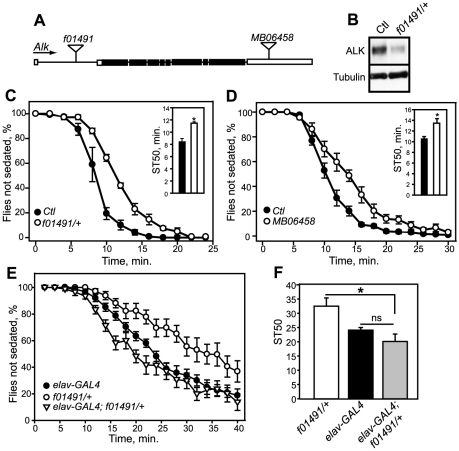
Insertions in *dAlk* affect ethanol sedation in flies. (A) Schematic of the *Alk* gene, showing the position of the *f01491* and *MB06458* P-element insertions. The boxes represent exons and connecting lines indicate introns. Shaded boxes illustrate the protein coding region. Arrow shows the direction of transcription. (B) Western blot showing reduced expression of dALK protein in heterozygous flies carrying the *f01491* insertion. Blot was stripped and probed with antibody to α-tubulin to demonstrate equal loading of total protein. (C, D) Ethanol sedation curves and ST50 graphs (inset) of flies carrying the *f01491* (C) and *MB06458* (D) insertions, illustrating increased resistance of *dAlk* mutant flies to ethanol-induced sedation. Error bars, SEM, n = 8. *P* = 0.002 (*f01491*) and *P* = 0.008 (*MB06458*), ANOVA. (E) Ethanol sedation curves of flies carrying the *f01491* insertion and *elav-GAL4c^155^*, showing rescue of *f01491* ethanol sedation resistance phenotype by re-expressing *dAlk* in neurons of the mutant. (F) ST50 values for the ethanol sedation curves in (E). *P = 0.0018 by ANOVA.

We next examined whether *dAlk* might modulate the flies' sensitivity to ethanol-induced sedation. A Flybase search revealed two P-element insertions in *Alk*, *f01491* (Exelixis Collection at the Harvard Medical School), located in a 5′ intron of the gene, and *MB06458* (Bloomington Drosophila Stock Center), located in the 3′ untranslated region (Figure 2A). Homozygous flies carrying the *f01491* insertion are not viable as adults and were therefore tested as heterozygotes. Flies carrying the *MB06458* insertion are homozygous viable and appear normal. To determine whether the insertions affect *dAlk* protein levels, we examined protein lysates from whole flies on western blots using an antibody to dALK [27]. Flies carrying the *f01491* insertion showed a 76% reduction in dALK compared to wild-type controls (Figure 2B). We did not observe a change in dALK levels in flies carrying the *MB06458* insertion (data not shown). We speculate that, since this is a weaker allele, the *MB06458* insertion may affect *Alk* expression in a subtle or more restricted, tissue-specific manner. Flies transheterozygous for *f01491*and *MB06458* were small and sickly and could therefore not be tested behaviorally; these data show, however, that the two P element insertions are allelic. We also crossed heterozygous flies carrying the *Alk^1^* allele [27], which expresses a truncated protein and is an ALK functional null, with heterozygous *f01491* mutant flies. We were unable to recover any transheterozygous *Alk^1^/f01491* mutants, indicating a failure of these mutations to complement.

We tested mutant flies for sensitivity to ethanol sedation using a modified loss-of-righting (LOR) assay and found that the *f01491* and *MB06458* insertions conferred increased resistance to ethanol sedation (Figure 2C, D). The time for 50% of the flies to become sedated (ST50) was calculated from the sedation curves by linear interpolation [5]. Flies carrying the *f01491* insertion sedated significantly later than control flies, with an ST50 of 11.5 min compared to 8.4 min (Figure 2C). Flies with the *MB06458* insertion also exhibited significantly delayed sedation with an ST50 of 13.5 min compared to 10.5 min for the control line (Figure 2D). The effect on ethanol sedation in these lines was not due to a difference in pharmacokinetics, as controls and mutant flies absorbed the same amount of ethanol (Figure S1). We next examined whether re-expressing *dAlk* in neurons of *f01491* mutant flies would rescue the ethanol sedation phenotype of the mutant. The *f01491* mutant flies were crossed to flies carrying *elav-GAL4^c155^* to drive neuronal expression of *dAlk* from the P-element insertion in the *f01491* mutant, which contains UAS sites in the orientation appropriate for GAL4-dependent transcription. The transcript expressed from the *f01491* insertion contains the entire protein-coding region of *dAlk*, since the P-element is located 5′ of the ORF (Figure 2A). *f01491* flies carrying the *elav-GAL4* transgene exhibited normal ethanol sensitivity implying a complete rescue of the behavioral phenotype (Figure 2E, F), and indicating that *dAlk* expression in neurons is sufficient for normal ethanol sedation sensitivity. These results suggest that the normal function of *dAlk* is to promote enhanced sensitivity to ethanol. The behavioral response of *dAlk* mutants to ethanol is consistent with the negative regulation of *dAlk* by dLMO, since we have found that the function of dLMO is to promote resistance to the sedating effect of ethanol [20].

### *Alk* expression correlates with behavioral responses to ethanol in mice

**Figure 3:**
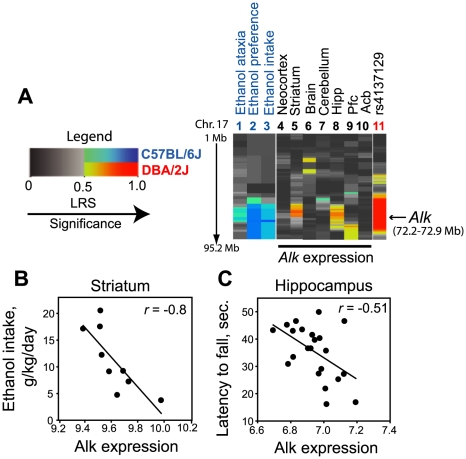
Putative regulation of ethanol-related behaviors and brain *Alk* expression by the *Alk* locus. (A) Heat map of mouse chromosome 17 (right panel) depicting correlations between ethanol-related traits (lanes 1–3), *Alk* expression in specific brain regions (lanes 4–10) and genotype at the *Alk* locus (lane 11). Legend for the heat map is shown on the left. Blue indicates that the C57BL/6J genotype at the locus is associated with increased expression of the trait, red indicates the same for DBA/2J genotype, and increased likelihood ratio statistic (LRS) is indicated by darker color. The red region surrounding the marker rs4137129 in the *Alk* locus (lane 11) is arbitrarily colored and indicates the extent of linkage disequilibrium. (B) Scatter plot depicting the correlation between *Alk* expression in the striatum and ethanol intake. Lower *Alk* expression was associated with increased ethanol intake. (C) Scatter plot depicting the correlation between hippocampal *Alk* expression and the latency to fall in seconds as a measure of ethanol-induced ataxia using the screen test. Lower *Alk* expression was correlated with increased latency to fall.

We hypothesized that *Alk* may regulate behavioral responses to ethanol in mammals since the expression of murine *Alk* is negatively regulated by LMO4 (Lasek *et al.*, submitted) and *Lmo3* and *Lmo4* play roles in regulating ethanol and cocaine-related behaviors in mice [19], [20]. Using the databases available in GeneNetwork (www.genenetwork.org), we tested the genotype of BXD recombinant inbred (RI) strains at a polymorphic marker in the *Alk* locus, rs4137129, for associations with two types of measures: gene expression in specific brain regions and ethanol-related behaviors (see Materials and Methods for a description of the analysis). We identified three traits in BXD recombinant inbred mice that differed by genotype at the *Alk* locus and correlated with variation in *Alk* expression in at least one brain region. These traits were ethanol-induced ataxia [28] and two highly inter-correlated measures of ethanol drinking, preference and intake [29]. The genetic influence on these traits is shown as a heat-map for chromosome 17 (Figure 3A), which also depicts the location of the *Alk* locus and apparent *Alk* cis-regulation of expression in two brain regions, the hippocampus and striatum. *Alk* expression in the hippocampus and striatum is higher in strains with the DBA/2J genotype at the *Alk* locus. Moreover, higher *Alk* expression in striatum strongly correlates with decreased ethanol intake (Figure 3B), whereas higher *Alk* expression in the hippocampus correlates with increased sensitivity to the ataxic effect of ethanol (Figure 3C). Together, these data suggest that *Alk* may be involved in behavioral responses to ethanol in mice.

### *Alk* regulates ethanol sedation in mice

**Figure 4:**
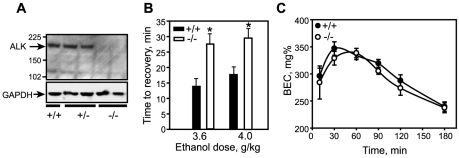
*Alk^KO^* mice show increased sedation in response to ethanol. (A) Western blot indicating loss of full-length ALK protein in the striatum of *Alk^KO^* mice (−/−) compared to heterozygous *Alk^KO^* (+/−) and wild-type (+/+) mice. Blot was stripped and probed with antibody to GAPDH to indicate equal protein loading. (B) LORR in *Alk^KO^* and wild-type mice at 3.6 and 4.0 g/kg ethanol. Shown is the time to recovery from sedation. *3.6 g/kg, F_1,24_ = 10.54, *P* = 0.003; *4.0 g/kg, F_1,28_ = 8.98, *P* = 0.006. (C) Blood ethanol concentration in *Alk^KO^* mice after an injection of 4.0 g/kg ethanol indicating no difference compared to wild-type controls. Shown is the blood ethanol concentration (BEC) in mg% over time. Error bars, SEM.

To test directly whether *Alk* is involved in regulating behavioral responses to ethanol, we generated *Alk* knockout (*Alk^KO^*) mice by targeting exons 20–21, which encode the juxtamembrane and N-terminal portion of the tyrosine kinase domain (Figure S2). *Alk^KO^* mice are fertile, viable, and appear normal, consistent with an independently generated *Alk^KO^* line which targets exon 22 and results in a truncated transcript [16]. We were also unable to discern any gross locomotor defects in the *Alk^KO^* mice, either under naïve conditions or in response to a saline injection (data not shown). We examined ALK protein expression in the striatum (specifically, nucleus accumbens) of our *Alk^KO^* mice and confirmed a loss of full-length ALK protein (Figure 4A), suggesting that we have generated a loss-of-function mutant similar to that described in Bilsland *et al.* [16]. We next examined *Alk^KO^* mice for their behavioral response to ethanol in a loss of righting reflex (LORR) test. Male and female wild-type and homozygous *Alk^KO^* mice were tested at two sedating doses of ethanol, 3.6 and 4.0 g/kg. At each dose of ethanol, we observed significant effects of genotype, but no effects of sex or sex by genotype interactions. Since we observed no effect of sex, we combined the LORR data for male and female mice (Figure 4B). ANOVA of the combined data demonstrated that *Alk^KO^* mice show a significant increase in the amount of time to recover the righting reflex at both doses of ethanol. Heterozygous *Alk^KO^* mice were also tested and did not show a difference compared to wild-type controls (data not shown). To determine if the difference in the LORR recovery time was due to alterations in ethanol metabolism and clearance in the *Alk^KO^* mice, we examined blood ethanol concentrations (BEC) at various time points after an injection of 4.0 g/kg ethanol (Figure 4C). No differences in the BEC were observed between genotypes. These data indicate that *Alk* negatively regulates ethanol-induced sedation time without affecting ethanol metabolism and clearance in mice.

### *Alk* regulates ethanol intake in mice in a binge drinking model

**Figure 5:**
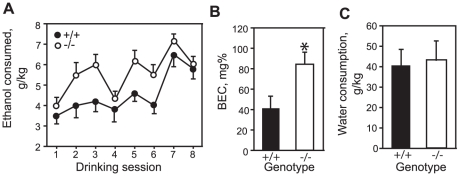
Increased ethanol consumption in *Alk^KO^* mice. (A) Ethanol consumption in wild-type (+/+) and homozygous mutant (−/−) *Alk^KO^* mice, expressed in g/kg over a 4-hour period for 8 drinking sessions in the dark. There was a significant effect of genotype (genotype: F_1,119_ = 7.71, *P* = 0.013; session:F_7,119_ = 10.19, *P*<0.001; genotype by session interaction: F_7,119_ = 1.12, *P* = 0.356). (B) Blood ethanol concentration (BEC, mg%) in wild-type and homozygous *Alk^KO^* mice after the final drinking in the dark session, indicating increased BEC in *Alk^KO^* mice. *P = 0.02. (C) Water consumption, expressed in g/kg over a 4-hour period for one drinking session in the dark, indicating no effect of genotype on general fluid intake. Error bars, SEM.

Since *Alk* expression in the striatum is negatively correlated with ethanol intake across the BXD RI strains (Figure 3B), we hypothesized that *Alk^KO^* mice would consume more ethanol than control mice. We tested ethanol intake in a limited intermittent-access drinking in the dark paradigm adapted from Rhodes *et al.* [30], in which mice drink to intoxication. Male wild-type and homozygous *Alk^KO^* mice were examined for consumption of a 20% ethanol solution over a 4-hour period 3 times per week during the dark cycle, for a total of 8 sessions. We observed a significant effect of genotype and drinking session, but no genotype by session interaction. This analysis indicates that all mice escalated their intake of ethanol after repeated sessions and that *Alk^KO^* mice consumed more ethanol overall than wild-type controls under these conditions (Figure 5A). Heterozygous male *Alk^KO^* mice were also tested and did not show a significant difference in ethanol intake when compared to wild-type controls (data not shown). An advantage of testing mice in the dark phase under limited access conditions is that mice will drink to intoxication. To confirm that the *Alk^KO^* mice drank intoxicating levels of alcohol, we tested the BEC of mice immediately following the final drinking session. Mice had blood ethanol levels greater than 0.04% (Figure 5B). Moreover, the *Alk^KO^* mice reached BEC levels significantly higher (>0.08%) than wild-type controls. To determine whether loss of *Alk* function might generally affect fluid consumption, we tested *Alk^KO^* mice for water intake using the same conditions used for ethanol consumption. No differences were observed between wild-type controls and *Alk^KO^* mice (Figure 5C). In conclusion, *Alk* negatively regulates alcohol consumption in mice without affecting general fluid consumption, suggesting that the normal function of *Alk* may be to curb excessive alcohol intake.

### Polymorphisms in human *ALK* are associated with the level of response to ethanol

We next explored the sequence variation in human *ALK* to determine if polymorphisms in this gene may be correlated with alcohol responses in humans. We sequenced the entire coding sequence of *ALK* (29 exons) in 348 subjects, all of whom had their response to an oral alcohol challenge measured in a laboratory setting [31]. After rapidly consuming approximately three drinks, the level of response (LR), representing each subject's reaction to alcohol, was measured every 30 minutes. Two LR tests were performed, motor coordination as measured by anterior/posterior body sway (BSA) and lateral body sway (BSL), and subjective “high” feeling using the Subjective High Assessment Scale (SHAS). All polymorphisms discovered through sequencing were then tested by regression for correlation between genotype and quantitative LR measures taken at 60 minutes post alcohol ingestion, the time-point when alcohol LR maximizes (see Materials and Methods for details).

Sequencing *ALK* in 348 subjects resulted in the discovery of 15 single nucleotide polymorphisms (SNPs). Nine of these SNPs were previously identified (dbSNP build 130) and the remaining 6 SNPs, all of which have a minor allele frequency (<1%), are newly discovered. For analysis, 7 SNPs with a minor allele frequency (MAF) of less than 1% were eliminated because markers with such a low MAF have essentially no power to detect association and would serve only to obscure potential associations with more informative markers if they are included in multiple test corrections.

**Figure 6:**
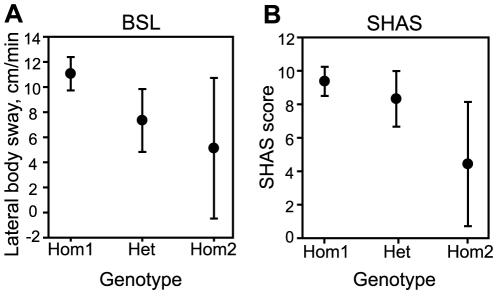
Effect of *ALK* genotype at rs17007646 on behavioral responses to alcohol in human subjects. (A) Body sway in the lateral direction (BSL) in cm/min as a function of genotype, indicating decreased BSL in response to alcohol in heterozygous individuals and individuals homozygous for the minor allele. (B) Subjective High Assessment Scale (SHAS) score in response to an alcohol challenge as a function of genotype. Individuals homozygous for the minor allele report a lower SHAS score. Shown are the group means. Error bars, SEM. Hom1, subjects homozygous for the major allele; Het, heterozygous subjects; Hom2, subjects homozygous for the minor allele.

The analysis uncovered 4 markers that are associated with at least one of the LR phenotypes at a nominal significance level (*P*<0.05). Two of these markers are associated with BSL with a false discovery rate (FDR) q-value of <0.05 (Table 1). Because of linkage disequilibrium among these markers (data not shown) it is likely that the different markers are not uncovering independent associations; rather, the data are consistent with a single genetic factor affecting alcohol LR that is in linkage disequilibrium with all four SNPs. We next explored in more detail the genotypic effect of one SNP, rs17007646, on two LR phenotypes, BSL and SHAS. Subjects heterozygous and homozygous for the minor allele at rs17007646 demonstrated reduced BSL in response to alcohol when compared to the major allele (Figure 6A). Similarly, subjects homozygous for the minor allele at rs17007646 reported a lower SHAS score than subjects homozygous for the major allele (Figure 6B). These data suggest that the minor allele at rs17007646 is associated with decreased sensitivity to alcohol in two behavioral measures, body sway and subjective high.

**Table 1:** Polymorphisms in human *ALK* significantly associated with altered behavioral responses to alcohol.

SNP ID	MAF	SNP Position	Amino Acid	Phenotype	Genotypes Tested	n	Value	*p*-value	q-value
rs56132472	0.10	Exon 29	T1446T	BSL	HOM1 vs. HET	329	0.39	0.0011	0.0310
rs17007646	0.13	Intron 28	non-coding	BSL	HOM1 vs. HET	330	0.33	0.0028	0.0387
rs1881421	0.42	Exon 29	D1529E	BSL	HOM1 vs. HET	323	0.24	0.0126	0.1159
rs17007646	0.13	Intron 28	non-coding	BSA	HOM1 vs. HOM2	329	0.48	0.0255	0.1245
rs56132472	0.10	Exon 29	T1446T	BSA	HOM1 vs. HET	328	0.25	0.0269	0.1245
rs4622670	0.30	Intron 20	non-coding	SHAS	HOM1 vs. HOM2	326	−0.40	0.0270	0.1245
rs17007646	0.13	Intron 28	non-coding	SHAS	HOM1 vs. HOM2	333	0.53	0.0434	0.1716

Columns (from left to right) show the single nucleotide polymorphism identifier (**SNP ID**), minor allele frequency (**MAF**), the position of the polymorphism in *ALK* (**SNP position)**, the amino acid change in the *ALK* protein (**Amino Acid**), the associated alcohol-related phenotype (**Phenotype**), the genotypic classes compared for association (**Genotypes Tested**), the number of subjects in the statistical test (**n**), the quantitative effect of being homozygous for the common allele vs. the other genotypic classes in the previous column, expressed in standard deviations (**Value**), the *p*-value rejecting the null hypothesis (genotype does not affect phenotype, ***p*** **-value**), and the FDR **q-value** multiple test correction.

## Discussion

The evidence presented here indicates that *Alk* contributes to behavioral responses to ethanol in invertebrates and mammals, including humans. Moreover, we found that *dAlk* expression is regulated by dLMO. LMO proteins are transcriptional regulators that function to repress or activate transcription through interactions with DNA binding proteins [26]. dLMO appears to repress *dAlk* expression, since *dAlk* increases in *dLmo* loss-of-function mutants. The ethanol sedation phenotype of *dLmo* and *dAlk* mutants is consistent with a negative regulatory role of dLMO on *dAlk* expression. The *dLmo EP1306* mutant, in which *dAlk* expression is increased, showed increased sensitivity to ethanol sedation [20], whereas the *dAlk f01491* mutant, with decreased *dAlk* expression, was resistant to ethanol sedation. These data define a novel regulatory pathway involved in ethanol-induced sedation in *Drosophila melanogaster*.

We observed phenotypic similarities between flies, mice, and humans in the acute response to alcohol as a function of genotype at the *Alk* locus. In the fruit fly, hypomorphic alleles of *dAlk* caused resistance to the motor incoordinating and sedating effects of ethanol. In mice, a polymorphism in *Alk* that is associated with low expression in the hippocampus also correlates with resistance to ethanol-induced ataxia. In humans, SNPs in *ALK* are associated with resistance to ethanol-induced ataxia as measured by body sway in response to an alcohol challenge. Insofar as motor-incoordinating behaviors related to ethanol can be compared between species, these results suggest that reduced *Alk* expression and/or function may result in resistance to the ataxic effect of ethanol. Interestingly, we found that *Alk^KO^* mice spent more time sedated in response to ethanol, suggesting that complete loss of *Alk* function leads to increased sensitivity to ethanol in this particular behavioral measure. The LORR test measures the length of time mice spend sedated and does not correlate with ethanol-induced ataxia in different strains of inbred mice [32]. Our data suggests that *Alk* differentially regulates ethanol-induced ataxia and LORR in mice. It is also possible that developmental compensation occurs in the *Alk* null mutant to alter sedation time in the LORR test; alternatively, this effect may be due to a brain-region specific defect not uncovered in BXD mice.

In addition to a role for *Alk* in the acute response to ethanol, we found that *Alk* regulates ethanol consumption in mice. Not only does reduced *Alk* expression in the striatum of BXD recombinant inbred mice correlate with increased ethanol intake in a two-bottle choice protocol, but global loss of ALK function (in *Alk^KO^* mice) leads to increased ethanol consumption in a limited-access binge drinking paradigm. We hypothesize that reduced *ALK* expression or function in humans (perhaps in individuals carrying minor alleles) might lead to increased ethanol consumption. Although not directly addressed in the current studies, low LR to ethanol in humans is correlated with increased risk for developing AUDs [2], [33]. We found 4 SNPs in *ALK*, all in linkage disequilibrium, that are associated with a low LR to ethanol in at least one of two behavioral measures, subjective high assessment and body sway, suggesting that *ALK* expression or function may be involved in the LR to ethanol and future development of AUDs. Only one of these SNPs, rs1881421, results in an amino acid change (Asp 1529 to Glu) located in the C-terminal region of the protein. This amino acid is not conserved between flies, mice, and humans and its role in ALK protein function is unknown. Determining the causative mutation(s) in *ALK* that may be involved in the decreased LR to ethanol is an area for future investigation.

How might signaling through ALK regulate behavioral responses to ethanol? In flies and mammalian cells in culture, ALK is known to activate the MAPK/ERK pathway [27], [34], [35], [36], [37], [38], [39]. We previously demonstrated that increased ERK signaling, through the receptor tyrosine kinase EGFR, in *Drosophila* neurons results in resistance to ethanol-induced sedation [5]. If ALK similarly stimulates ERK phosphorylation, then one would predict that loss of *dAlk* function would lead to decreased ERK phosphorylation and increased sensitivity to ethanol-induced sedation. However, the results described here indicate that reducing ALK levels instead leads to increased resistance to the sedating effect of ethanol in *Drosophila*. In an attempt to discern whether ERK phosphorylation is altered with loss of ALK function, we examined ERK phosphorylation in the heads of mutant *f01491* flies by western blotting. We did not observe any changes in ERK under these conditions (AWL, CLK and UH, unpublished results). It is possible that levels of phosphorylated ERK are altered in a subset of neurons in the brain that we could not detect with western blotting of whole heads, or that changes in ERK phosphorylation are too subtle to detect by western blotting. Alternatively, ethanol treatment may be required to determine if ERK phosphorylation is altered in *dAlk* mutants. Another hypothesis is that *dAlk* may signal through a different pathway, such as JAK/STAT or PI3K to affect ethanol-induced sedation in *Drosophila* [40].

In a related experiment in mice, we examined levels of phosphorylated MEK, an upstream activator of ERK, in the striatum of ethanol-naïve *Alk^KO^* mice. Interestingly, we found a 41% increase in MEK phosphorylation in this brain region (Figure S3). Increased expression of genes in the ERK pathway has been demonstrated in mouse lines selectively bred for high ethanol-preference [41]. Our data in the *Alk^KO^* mice might suggest that increased ERK signaling could be responsible for the increased ethanol consumption in these mice. Whether ALK normally inhibits ERK activation in the striatum, or whether increased MEK phosphorylation is due to compensatory changes in the *Alk^KO^* mice is a subject for future investigation. Our data, combined with the findings in ethanol-preferring mice, suggest that treatment with MEK inhibitors might be a potential therapeutic strategy for excessive ethanol consumption and AUDs. Faccidomo *et al.* tested the MEK inhibitor SL 327 for ethanol self-administration in C57BL/6 mice and observed a biphasic response to MEK inhibition [42]. Low doses increased operant responding for ethanol, while high doses decreased responding. Ethanol induces c-fos expression, an effect that is suppressed in alcohol-dependent animals [43]. This suppression is alleviated by the MEK inhibitor UO126 only in dependent animals, suggesting a complicated relationship between MEK activity and alcohol dependence.

Interestingly, the *ALK* marker rs1881421 in humans, which appears to be associated with body sway in response to an alcohol challenge (Table 1), is also associated with schizophrenia in a Japanese population [17], implicating *ALK* in other neuropsychiatric diseases beyond AUDs. In addition, we have discovered that *Alk* regulates behavioral responses to cocaine in mice (Lasek *et al.*, submitted); *Alk* may therefore regulate behavioral responses to multiple drugs of abuse. Future studies will focus on the neural and molecular mechanisms of *Alk* function in order to gain a greater understanding of its role in AUDs and other neuropsychiatric disorders.

## Materials and Methods

### Ethics Statement

Human subjects that had DNA re-sequenced were part of the San Diego Sibling Pair Investigation [31], [44], who underwent written informed consent procedures as approved by the University of California, San Diego Human Subjects Protections Committee. All animal protocols were in strict accordance with the recommendations in the Guide for the Care and Use of Laboratory Animals of the National Institutes of Health and approved by the Ernest Gallo Clinic and Research Center Institutional Animal Care and Use Committee (approval number 09.11.198). The Gallo Center is accredited by the Association for Assessment and Accreditation of Laboratory Animal Care (AAALAC) International.

### *Drosophila* Culture and Strains

All flies were maintained on standard cornmeal and molasses agar food at 25°C and 70% humidity. The *Bx^J^* mutant has been described previously [24]. The *dLmo* loss-of-function mutants *EP1306*, *Hdp[R26]* and *Pdrm[5–73]* have been described previously [18]. Lines were out-crossed for at least five generations to a *w^1118^* stock isogenic for Chromosomes II and III; these “*w*; iso” flies served as the genetic background control for quantitative PCR and microarray experiments. The *dAlk* P-element insertion mutant *MB06458* was obtained from the Bloomington Drosophila Stock Center (Bloomington, IN, USA) and *f01491* was obtained from the Exelixis Collection at the Harvard Medical School. The presence of each insertion in the *Alk* locus was confirmed by PCR. Lines were outcrossed for at least 5 generations to *white Berlin* (*f01491*) or *2202U* (*MB06458*). The *white Berlin* and *2202U* strains served as genetic background controls for the corresponding mutant lines in behavioral assays. All experiments used adult male flies which were collected 2–4 days following eclosion.

### Gene Expression Microarrays

All microarray data are MIAME compliant. Raw data has been deposited in NCBI's Gene Expression Omnibus and are accessible through GEO Series accession number GSE25988. RNA was isolated from 3 groups of 200 fly heads from each genotype and hybridized to Affymetrix Drosophila 2.0 oligonucleotide microarray chips at the Partners HealthCare Center for Personalized Genetic Medicine Microarray Facility (Harvard University). Microarray data was processed and analyzed as described in Kong *et al.* [45]. Genes were selected as differentially expressed by pairwise t-tests between the *w;iso* control, the *Bx^J^*, and the *EP1306* mutants with a cutoff of *P*<0.05 following normalization and subtraction of the averaged *w;iso* control. The 555 genes that were chosen for clustering were then further selected by ANOVA with a *P*<0.05. Clustering was performed using the HOPACH (Hierarchical Ordered Partitioning and Collapsing Hybrid) algorithm [25].

### Real-time Quantitative PCR

Total RNA isolation from whole flies using TRIzol® (Life Technologies, Carlsbad, CA USA), cDNA synthesis, and real-time quantitative PCR were performed as described in Corl *et al.* [5]. RNA was isolated from 6–7 independent biological replicates of flies. Pre-designed 20× TaqMan® probe and primer mixes targeting *Alk* (Dm01797078_g1) and the ribosomal protein control transcript *RpL32* (Dm02151827_g1) were purchased from Life Technologies. Data was normalized using the standard curve method and assessed for statistical significance using ANOVA.

### *Drosophila* Behavioral Assays

Ethanol sedation assays were performed essentially as previously described [5], [46]. Samples of 25–30 male flies were allowed to equilibrate for ∼10 min to humidified air in the booz-o-mat apparatus [4] before starting exposure to a 100∶50 mixture of ethanol vapor∶humidified air (100∶50 E∶A). Ethanol exposure commenced at 0 min of the assay and was continuous thereafter. At 2-min intervals, each tube of flies (8 tubes per assay) was twirled and the number of flies that appeared unable to right themselves was scored by an experimenter blinded to sample identity. Typical assay duration was 30 min.

### Animals

*Alk^KO^* mice were backcrossed to C57BL/6J for 4 generations prior to behavioral testing. Heterozygous mice were intercrossed to generate wild-type and homozygous littermates for behavioral tests. Animals were group housed, unless they were tested for ethanol consumption, at which point they were singly-housed for two weeks prior to testing ethanol intake. Food and water were provided at all times and animals were on a 12 hour light-dark cycle.

### Generation of *Alk* knockout mice

An EcoRI fragment of the mouse *Alk* genomic DNA locus was used to prepare the *Alk* targeting construct, in which the two exons encoding the juxtamembrane domain and N-terminal portion of the kinase domain of *Alk* (exons 20 and 21, respectively) were replaced by a neomycin expression cassette. An EcoRI site was introduced into the targeted locus by the neomycin cassette to facilitate subsequent genotyping of targeted ES cells and mice. A herpes simplex virus–thymidine kinase gene cassette to enable negative ES cell selection with ganciclovir was inserted in the 5′ end of the *Alk*-neomycin construct. Electroporation of the linearized *Alk* targeting vector into E14 ES cells was done as described [47], [48]. Correctly targeted ES cell clones with normal karyotypes were injected into C57BL/6 blastocysts to generate chimeric mice for subsequent breeding to obtain germline transmission. Mice were genotyped by Southern blot analysis or PCR. *Alk^KO^* mice were genotyped by PCR using primers to *Alk* and the neomycin resistance gene (Primers, 5′-3′: *Alk* forward, ACCCCCTCACAGCGGACACCTATC; *Alk* reverse, TGGGGACAGGGGCAGATGATTGAC; Neomycin forward, ATCTCCTGTCATCTCACCTTGCTC; Neomycin reverse, GTAAAGCACGAGGAAGCGGTCAGC). Figure S2 shows a representative PCR genotyping.

### Western blots

For western blots on whole flies or fly heads, approximately 30 frozen flies or 100 heads were homogenized in 1 mL of RIPA buffer (10 mM Tris, pH 7.5, 150 mM NaCl, 1 mM EDTA, 1% nonidet P-40, 0.5% sodium deoxycholate, and 1% SDS) containing freshly added protease inhibitors (aprotinin, pepstatin, and phenylmethanesulfonyl fluoride). After homogenization, lysates incubated on ice for 1 hr, and were centrifuged at 4°C for 10 min. The supernatant was removed and treated for electrophoresis as described below. For western blots on mouse brain, punches of fresh nucleus accumbens tissue were frozen in liquid nitrogen. Tissue was thawed on ice and homogenized in 50 µL of RIPA buffer containing freshly added complete mini protease inhibitor tablet (Thermo Fisher Scientific, Asheville, NC). After clarifying lysates by centrifugation, a BCA protein assay (Thermo Fisher Scientific) was performed on samples. 4× LDS buffer (Life Technologies) containing β-mercaptoethanol was added to 20 µg of lysate. Samples were subjected to electrophoresis and western blotting using the NuPAGE® Novex® Tris-Acetate mini gels (Life Technologies) and the ECL Plus western blotting detection system (GE Healthcare, Piscataway, NJ) according to manufacturer's instructions. Western blots were probed with ALK-c antibody (for mouse ALK, ab650, Abcam, Cambridge, MA) diluted 1∶500 in 5% bovine serum albumin or with *Drosophila* ALK rabbit antibody [27] diluted 1∶5000 in 5% milk. Blots were probed with HRP-conjugated secondary antibodies (rabbit or mouse, GE Healthcare) diluted 1∶5000, then stripped using Restore Plus™ Western blot stripping buffer and re-probed with an antibody to GAPDH or Tubulin (Thermo Fisher Scientific).

### Loss of the Righting Reflex (LORR)

Male and female wild-type and homozygous *Alk^KO^* mice, aged 2–6 months were injected i.p. with 20% ethanol in saline (v/v) at a dose of 3.6 g/kg. After injection, each mouse was placed on its back and tested for the ability to right itself. The mouse was determined to have lost the righting reflex if it could not right itself 3 times within 30 sec. and regained the righting reflex if it could fully right itself 3 times within 30 sec. The duration of the loss of righting reflex was calculated as the difference between the time when the reflex was lost and when it was regained. One week after the first test, mice were tested again at a dose of 4 g/kg ethanol. Data were analyzed for statistical significance using ANOVA.

### Measurement of Blood Ethanol Concentration

Blood ethanol concentration (BEC) was measured in the same group of mice used in the LORR experiment three weeks after the final LORR test. Mice were injected with 4.0 g/kg of ethanol i.p. and 20 µL of blood was obtained via tail vein puncture at 10, 30, 60, 90, 120, and 180 min post-injection. Blood samples were stored at -80°C until BECs were determined using an NAD-ADH enzymatic assay [49]. BECs were also measured immediately after the 8^th^ session of the limited intermittent-access drinking experiment.

### Limited intermittent-access drinking in the dark

The intermittent-access drinking protocol was adapted from Rhodes *et al.* [30]. Ethanol-naive mice were singly housed in single grommet cages in a reverse light-dark cycle room (lights off from 10 a.m. to 10 p.m.) and allowed to acclimate for two weeks prior to the study. Following acclimation, home cage water bottles were replaced with a single bottle of 20% v/v ethanol in water at noon for 4 hours on Monday, Wednesday, and Friday, for a total of 8 ethanol sessions. Bottles were weighed before and after each session and mice were weighed once a week. Baseline water consumption was measured one day before the beginning of ethanol access by weighing a water bottle before and after a 4 hour session. Mice had *ad libitum* access to water when ethanol was not present. Ethanol consumption (g ethanol/kg mouse/4 hrs) was calculated as the difference between bottle weight before and after drinking sessions. Drinking volumes were corrected for spillage by subtracting weight lost from two control bottles of 20% ethanol placed on empty cages for the duration of the sessions. Data were analyzed for statistical significance by 2-way RM ANOVA.

### GeneNetwork methods and statistics

To examine whether *Alk* might be involved in the expression of ethanol-related behaviors, complementary database approaches similar to those described in Lu *et al.* were employed [50]. First, the genotypes of the BXD recombinant inbred (RI) strains at a marker in the *Alk* locus, rs4137129, were tested for associations with ethanol-related behaviors in GeneNetwork (www.genenetwork.org). Genotypic differences in the expression of the behavior were considered significant at *P*<0.05 by uncorrected t-tests for the comparison of C57BL/6J and DBA/2J genotype. Second, variation in *Alk* expression in multiple brain regions, including the whole brain [51], cerebellum, hippocampus [52], neocortex [53], nucleus accumbens, prefrontal cortex, and striatum, was examined for correlations with RI strain means for all of the available behaviors in GeneNetwork. We used a cutoff of |*r*|≥0.5 to identify potentially meaningful correlations between *Alk* expression and behavior. The traits identified by both techniques were then combined, resulting in a list of traits that differed as a function of genotype at the *Alk* locus and were associated with variation in *Alk* expression in at least one brain region.

### Human subjects and testing protocol

Recruited subjects met the following criteria: ages 18–29; had previously consumed alcohol but did not meet dependence criteria; had at least one parent with repetitive alcohol-related life problems who met the criteria for alcohol dependence using the Diagnostic and Statistical Manual of the American Psychiatric Association, 4th edition (DSM IV); and were part of a family where at least two siblings met the same criteria (although incomplete recruitment did yield many single sibling families). Recruited siblings were given an alcohol challenge by consuming a 20% v/v solution of 0.75 ml/kg of ethanol (0.6 g/kg for women, and 0.9 g/kg for men) within an 8-minute period. Doses were chosen to produce similar blood alcohol concentrations among individuals. At baseline, 15 min, 30 min, and every half hour after consuming the alcohol, subjects filled out the Subjective High Assessment Scale (SHAS) indicating their feelings of intoxication on 13 items, each rated on a 36-point scale indicating perceived subjective changes from baseline. Subjects were also tested for motor coordination by quantifying body sway over a 1 minute period (BSA-body sway anterior/posterior and BSL-body sway lateral).

### Genetic association analysis

In this analysis, the SHAS and BS scores at the time of peak alcohol effect (60 min) were used. 348 subjects were genotyped and tested for association. The subjects comprise 179 independent families: 34 single sibling families; 115 two sibling families; 21 three sibling families; 4 four sibling families. Phenotypes were corrected for non-normality using the Box-Cox transformation and scaled to mean = 0 and SD = 1. The tests of association were performed in R with the lmekin function of the kinship package. This function provides a linear mixed effects model whereby the genetic relatedness among individuals (based on the kinship coefficient) is incorporated into the covariance structure of the random effects. The fixed effect is used for the tests of association and adjustments for covariates. It included the covariate sex plus the test SNP (a factor of genotypes). Two contrasts were examined, each with the Wald test: the major homozygote (Hom1) against the heterozygote (Het); and the major homozygote against the minor homozygote (Hom2). Statistical tests were reduced by only testing markers with an MAF>1% and eliminating the Hom2 against the Het as the power of these tests is very low due to small numbers in one of the genotype classes. FDR q-values were calculated using the method of Storey and Tibshirani [54].

### DNA preparation

DNA was extracted from blood specimens within 5 days of collection. DNA was extracted using Gentra Puregene reagents and protocols (Qiagen, Valencia, CA USA). Extracted DNA was quantified using the Pico Green method (Life Technologies) and all stocks were normalized to a common concentration for sequencing assays.

### Human genomic re-sequencing and genotyping

All SNP discovery and genotyping was performed using fluorescent Sanger sequencing of PCR templates generated directly from a subject's genomic DNA. Primers flanking the *ALK* exons were used to directly isolate sequencing template from genomic DNA. Sequencing/genotyping was performed by fluorescent Sanger chemistry as implemented by Applied Biosystems (Life Technologies) directly applied to the PCR amplified *ALK* exons. The sequences generated were analyzed for the presence of SNPs and genotyped using the software package Mutation Surveyor from SoftGenetics LLC (State College, PA, USA). To maximize sensitivity and accuracy, all analysis was performed on both strands and all heterozygote calls were verified by human inspection.

## Supporting Information

Figure S1 **Flies carrying the** ***MB06458*** **insertion do not show altered ethanol absorption.** For each sample, 25 male control and *MB06458* flies were exposed for 15 min to ethanol at a dose of 100∶50 (ethanol vapor∶air) and immediately frozen. Ethanol concentration was measured using a NAD∶ADH enzymatic assay on homogenized flies. Shown is the % ethanol concentration obtained in each group of flies. Error bars, SEM, n = 5.
(EPS)

Figure S2 **Generation of** ***Alk^KO^*** **mice.** (A) Scheme for generating *Alk^KO^* mice. An EcoRI fragment of the *Alk* genomic DNA locus was used to develop the *Alk* targeting vector, in which the exons coding for juxtamembrane domain of the ALK protein and the ATP binding pocket of the kinase domain, and their flanking sequences were replaced by a neo expression cassette. An EcoRI site was introduced into the targeted locus by the neo cassette. A herpes simplex virus–thymidine kinase gene cassette mediating negative ES cell selection with ganciclovir was inserted in the 5′ end of the *Alk*-neo construct. Correctly targeted ES cell clones with normal karyotypes were injected into blastocysts to generate chimeric mice for subsequent breeding to obtain germline transmission. Letters indicate restriction enzyme sites (B) Example of PCR genotyping of the *Alk^KO^* mice. Shown is a representative agarose gel illustrating PCR product. Mice were genotyped using primers to *Alk* at the neo insertion site to determine the presence or absence of the wild-type allele and to neo to determine the presence or absence of the insertion (NEO). The genotype (+/+, +/−, −/−) based on the PCR is indicated above the gel, with a negative control (no template DNA) for PCR and the 100 bp molecular weight marker on the right side of the gel. Note the presence of a single PCR product indicates either wild-type (+/+) or homozygote (−/−), while the presence of both Alk and NEO PCR products indicates a heterozygote (+/−).
(EPS)

Figure S3 **Increased MEK phosphorylation in the striatum of** ***Alk^KO^*** **mice.** Striatum was dissected from the brains of adult male mice and analyzed using the PathScan® Phospho-MEK1 and Total MEK1 Sandwich ELISA kits from Cell Signaling Technology. Shown is the ratio of phosphorylated MEK to total MEK (pMEK/MEK) in wild-type (+/+) and *Alk^KO^* (−/−) mice. *P = 0.005 by Student's t-test, n = 5–7.
(EPS)

Table S1 **Transcripts showing significantly altered expression in** ***dLmo*** **mutants** ***EP1306*** **and** ***Bx^J^*** **.** Columns (from left to right) indicate the Affymetrix probe set identifier, the gene symbol, the FlyBase annotation, the cluster number from HOPACH clustering, the mean expression value of the transcript (normalized to expression in control *w;iso* flies and expressed as log_2_) in flies carrying the *BxJ* and *EP1306* mutations, and the ANOVA *p*-value.
(XLS)
